# Supplementary material for: The integration of technology into a home-based visuo-cognitive training intervention for people with Parkinson’s: Is the future digital?
Source: PLoS One. 2023 Jun 15;18(6):e0285100. doi: 10.1371/journal.pone.0285100 (PMC10270359; doi:10.1371/journal.pone.0285100)
Supplement: S1 File — (DOCX) [file pone.0285100.s001.docx]

| **Coding Framework** | |
| --- | --- |
| **Initial codes** | **Sub themes** |
| 1. **Background**    1. Personal philosophy   1.1.2 Attitude to PD  1.1.3 Outlook on life   - 1. Vision and PD      1. Personal experience      2. Prior knowledge   2. Reasons for taking part      1. Personal gain      2. Altruistic   3. Expectations of study   4. Perception of technology      1. Prior experience      2. “The bigger picture”      3. Avoids technology  1. **Standard intervention**    1. Pen and paper    2. Exercises    3. Engagement 2. **Using the technology**    1. Strobes       1. Length of training       2. Physical comfort       3. Barriers to use       4. Impact on exercise    2. App       1. Specific drills       2. Barriers       3. Facilitators 3. **Expressing preferences**    1. Favour of technology    2. Favour of standard approach    3. Undecided/mixed view    4. Personal factors       1. Age       2. Attitude       3. Physical ability 4. **Intention to use the technology**   5.1 Would use independently  5.2 Would not use beyond study  5.3 Need for support  5.3.1 Professional support  5.3.1 Support of “other”  5.4 Safety   1. **Outcomes**    1. Physical e.g. balance, gait    2. Mood/outlook    3. Energy levels    4. Negative effects    5. Confidence to exercise    6. Intention to exercise 2. **Impact of taking part in research**     1. Rewarding    2. Enjoyable    3. Personal gain    4. Integration with society    5. Interest in outcome | - Context - Understanding impact of visual problems of visual problems - Expectations - “Buy in” - Technology self-efficacy - Remote health - Compatibility (how technology fits with existing behaviour) - Added challenge - Technical challenges - Motivation - Responsiveness - Sense of achievement - Barriers to use - Relative advantage (superiority of one intervention over the other) - Sociodemographic factors affecting engagement - Physical factors (relating to PD) - Value of face-to-face contact - Safety - Intention to use - Physical outcomes - Impact on mood/energy levels - Behaviour change - Willingness to take part in future research |
