# Supplementary material for: The integration of technology into a home-based visuo-cognitive training intervention for people with Parkinson’s: Is the future digital?
Source: PLoS One. 2023 Jun 15;18(6):e0285100. doi: 10.1371/journal.pone.0285100 (PMC10270359; doi:10.1371/journal.pone.0285100)
Supplement: Attachment — Submitted filename: Response to reviewers.docx [file pone.0285100.s002.docx]

**PONE-D-22-33798: The integration of technology into a home-based visuo-cognitive training intervention for people with Parkinson’s: is the future digital?**

We would like to thank the editors and the reviewers for their hard work in reviewing our manuscript. We have added our reviewer responses below. All revisions are identified in the document: “Revised manuscript with tracked changes”.

| **Editors requirements** | **Authors’ response** |
| --- | --- |
| 1. Please ensure that your manuscript meets PLOS ONE's style requirements, including those for file naming. The PLOS ONE style templates can be found at  <https://journals.plos.org/plosone/s/file?id=wjVg/PLOSOne_formatting_sample_main_body.pdf> and  <https://journals.plos.org/plosone/s/file?id=ba62/PLOSOne_formatting_sample_title_authors_affiliations.pdf> | Following PLOS ONE style requirements, we have removed author qualifications from the title page and ensured that all headings are written in sentence case throughout the main body of the manuscript. |
| 2. Please note that PLOS ONE has specific guidelines on code sharing for submissions in which author-generated code underpins the findings in the manuscript. In these cases, all author-generated code must be made available without restrictions upon publication of the work. Please review our guidelines at <https://journals.plos.org/plosone/s/materials-and-software-sharing#loc-sharing-code>  and ensure that your code is shared in a way that follows best practice and facilitates reproducibility and reuse. | We have included our coding framework uploaded in S1 File and have referenced this in the manuscript (line 168 of the unmarked manuscript). |
| 3. Thank you for stating in your Funding Statement:  "This study is funded by a Northumbria University PhD studentship (JD) in collaboration with Senaptec Inc. (Beaverton, Oregon, USA) (PI: SS). SS is supported, in part, by a Parkinson’s Foundation Post-Doctoral Fellowship for Basic Scientists (PF-FBS-1898-18-21) and a Clinical Research Award (PF-CRA-2073)."  Please provide an amended statement that declares *all* the funding or sources of support (whether external or internal to your organization) received during this study, as detailed online in our guide for authors at <http://journals.plos.org/plosone/s/submit-now>.  Please also include the statement “There was no additional external funding received for this study.” in your updated Funding Statement.  Please include your amended Funding Statement within your cover letter. We will change the online submission form on your behalf. | We have amended our Funding Statement and included the amended version within our cover letter as requested:  "This study is funded by a Northumbria University PhD studentship (JD) in collaboration with Senaptec Inc. (Beaverton, Oregon, USA) (PI: SS) and a small British Geriatric Society Movement Disorder Special Interest Group research grant. SS is supported, in part, by a Parkinson’s Foundation Post-Doctoral Fellowship for Basic Scientists (PF-FBS-1898-18-21) and a Clinical Research Award (PF-CRA-2073). There was no additional external funding received for this study." |
| 4. Thank you for stating the following financial disclosure:  "This study is funded by a Northumbria University PhD studentship (JD) in collaboration with Senaptec Inc. (Beaverton, Oregon, USA) (PI: SS). SS is supported, in part, by a Parkinson’s Foundation Post-Doctoral Fellowship for Basic Scientists (PF-FBS-1898-18-21) and a Clinical Research Award (PF-CRA-2073). "  Please state what role the funders took in the study. If the funders had no role, please state: ""The funders had no role in study design, data collection and analysis, decision to publish, or preparation of the manuscript.""  If this statement is not correct you must amend it as needed.  Please include this amended Role of Funder statement in your cover letter; we will change the online submission form on your behalf. | We have included the following statement in our cover letter as requested:  “We can confirm that the funders had no role in study design, data collection and analysis, decision to publish, or preparation of the manuscript.” |
| 5. We note that you have indicated that data from this study are available upon request. PLOS only allows data to be available upon request if there are legal or ethical restrictions on sharing data publicly. For more information on unacceptable data access restrictions, please see <http://journals.plos.org/plosone/s/data-availability#loc-unacceptable-data-access-restrictions>.  In your revised cover letter, please address the following prompts:  a) If there are ethical or legal restrictions on sharing a de-identified data set, please explain them in detail (e.g., data contain potentially sensitive information, data are owned by a third-party organization, etc.) and who has imposed them (e.g., an ethics committee). Please also provide contact information for a data access committee, ethics committee, or other institutional body to which data requests may be sent.  b) If there are no restrictions, please upload the minimal anonymized data set necessary to replicate your study findings as either Supporting Information files or to a stable, public repository and provide us with the relevant URLs, DOIs, or accession numbers. For a list of acceptable repositories, please see <http://journals.plos.org/plosone/s/data-availability#loc-recommended-repositories>.  We will update your Data Availability statement on your behalf to reflect the information you provide. | We have included the following statement in our cover letter:  “The minimal data needed to support our study results have been integrated in the “Data analysis” and “Results” sections of the paper. To assure and protect participant’s privacy, the full datasets (interview transcriptions) are not available. Due to the qualitative methodology adopted in the study (including the small sample size, inclusion of individual participant characteristics and the defined geographical region), interviews may contain sensitive information that could lead to participants identification. The authors have assured data privacy and confidentiality to participants before enrolling on the study, assuring that interview transcripts would only be overseen for research purposes by the authors.” |
| 6. We note that you have stated that you will provide repository information for your data at acceptance. Should your manuscript be accepted for publication, we will hold it until you provide the relevant accession numbers or DOIs necessary to access your data. If you wish to make changes to your Data Availability statement, please describe these changes in your cover letter and we will update your Data Availability statement to reflect the information you provide. | We would like to make amendments to our data availability statement following review of our ethics approval and have included the following statement in our cover letter:  *“Relevant excerpts could be made available upon request but only those that have been anonymised. The ethical approval process was dependent upon no raw data being shared outside of the research team and we have not received ethical approval to store excerpts in a data repository. This study was approved by Northumbria University’s ethical approval system. Data requests can be sent to dp.officer@northumbria.ac.uk.”* |
| 7. We note that Figure (Table 1) in your submission contain copyrighted images. All PLOS content is published under the Creative Commons Attribution License (CC BY 4.0), which means that the manuscript, images, and Supporting Information files will be freely available online, and any third party is permitted to access, download, copy, distribute, and use these materials in any way, even commercially, with proper attribution. For more information, see our copyright guidelines: <http://journals.plos.org/plosone/s/licenses-and-copyright>.   We require you to either (1) present written permission from the copyright holder to publish these figures specifically under the CC BY 4.0 license, or (2) remove the figures from your submission:   a. You may seek permission from the original copyright holder of Figure (Table 1) to publish the content specifically under the CC BY 4.0 license.    We recommend that you contact the original copyright holder with the Content Permission Form (<http://journals.plos.org/plosone/s/file?id=7c09/content-permission-form.pdf>) and the following text: “I request permission for the open-access journal PLOS ONE to publish XXX under the Creative Commons Attribution License (CCAL) CC BY 4.0 (<http://creativecommons.org/licenses/by/4.0/>). Please be aware that this license allows unrestricted use and distribution, even commercially, by third parties. Please reply and provide explicit written permission to publish XXX under a CC BY license and complete the attached form.”   Please upload the completed Content Permission Form or other proof of granted permissions as an ""Other"" file with your submission.    In the figure caption of the copyrighted figure, please include the following text: “Reprinted from [ref] under a CC BY license, with permission from [name of publisher], original copyright [original copyright year].”   b. If you are unable to obtain permission from the original copyright holder to publish these figures under the CC BY 4.0 license or if the copyright holder’s requirements are incompatible with the CC BY 4.0 license, please either i) remove the figure or ii) supply a replacement figure that complies with the CC BY 4.0 license. Please check copyright information on all replacement figures and update the figure caption with source information. If applicable, please specify in the figure caption text when a figure is similar but not identical to the original image and is therefore for illustrative purposes only. | Thank you for highlighting this. We have removed the original images from Table 1 and have supplied replacement images that comply with the CC BY 4.0 license. |
| 8. Please review your reference list to ensure that it is complete and correct. If you have cited papers that have been retracted, please include the rationale for doing so in the manuscript text, or remove these references and replace them with relevant current references. Any changes to the reference list should be mentioned in the rebuttal letter that accompanies your revised manuscript. If you need to cite a retracted article, indicate the article’s retracted status in the References list and also include a citation and full reference for the retraction notice. | We are not aware that any papers from our reference list have been retracted. |

**Reviewer #1 Comments**: *This paper examined the experiences of individuals with Parkinson's disease while using an at-home visuo-cognitive training program. The authors used Normalization Process Theory to evaluate the subjective experience the individuals had with the program. The primary goal was to evaluate potential factors that would drive use of this program from the end-user perspective. The research subjects were a subset of a larger randomized controlled trial. Overall the paper is well-written and interesting, although is a small sample and preliminary. This initial evaluation of the program/technology is an important part of the developmental process. The authors provided a detailed description of the analysis/theory which is appreciated. Below are some minor suggestions to improve the quality of the manuscript.*

| **Reviewer #1 comment** | **Authors’ response** |
| --- | --- |
| Pg. 3, line 67: Although the technology is outlined in Table 1, it would be helpful to add a general overview of the training in the introduction. | Thank you for this suggestion. We have directed the reader to our protocol paper on page 3 lines 67-68 which has a full description of both the TVT and standard interventions:  *“For full information on the TVT intervention described in this study, please see our previously published study protocol (Das et al. 2022)”* |
| Pg. 3, line 68: please define these "gaps", it will help to support your "WHY" this is needed. | Thank you for this suggestion. We have included the following text to provide more context to the reader:  “*These gaps are in part due to the digital immaturity of healthcare systems, but are also a result of limitations in “real-world” research where technological solutions are developed without consideration of how they will be received by service users(16*)” |
| Results: There is some repetitions between the tables and the text-- especially in subject quotes. Please make sure that these statements are presented only once. | Thank you for highlighting this. We have removed participant quotations from both the tables and main body of text where there was repetition of these data. |
| Although this was a qualitative study, it would be helpful to add some data about trends in subject statements, similar to what was noted on line 200 -- the majority of the subjects (give numbers to support this statement, for example 6 out of 8) | Where we used the term “majority of participants” in the main body of the manuscript, we have provided specific numbers to support the statement as suggested. |
| Table 5: Please remove extra lines in this table to make it cleaner. | We have revised the table to reduce the additional lines in the headers. Clarification of abbreviations is now included in the footnotes below the table. |
| Overall there are a few typos and grammatical errors- please review for those. | We have reviewed the manuscript for typos and grammatical errors and have corrected these with tracked changes in the main body of the text. We acknowledge that there are some grammatical errors within the participant quotations, but we have chosen to keep these in as they have been transcribed verbatim. For example, line 202 of the umarked manuscript:  *“… there is parts of the day when my eyesight is worse...”* |

**Reviewer #2 comments***: I really enjoyed this article - it was a well written qualitative study. I honestly have so few suggestions that I'm almost embarrassed as a reviewer! The only comment I have is just to double check consistency in format of headings - sometimes all words are capitalized, sometimes only the first is. That's such a minor thing, but the one thing I caught. From a methodological standpoint, it was great. The authors noted the limitation of the small sample, but it was also appropriate for the design. Well done, all around.*

We would like to thank Reviewer #2 for their positive feedback and kind words. Thank you for highlighting the inconsistencies in formatting. We have revised the headings to ensure consistency throughout the manuscript.
